# Clinical value of a comprehensive clinical- and echocardiography-based risk score on predicting cardiovascular outcomes in ischemic heart failure patients with reduced ejection fraction

**DOI:** 10.1007/s00392-024-02399-1

**Published:** 2024-03-06

**Authors:** Dan Liu, Kai Hu, Camilla Wagner, Björn Daniel Lengenfelder, Georg Ertl, Stefan Frantz, Peter Nordbeck

**Affiliations:** 1 Department of Internal Medicine I - Cardiology, University Hospital Würzburg, Oberdürrbacher Str. 6, 97080 Würzburg, Germany; 2 Comprehensive Heart Failure Center, Würzburg, Germany

**Keywords:** Ischemic heart failure, Left ventricular ejection fraction, Risk stratification, Prediction model, Echocardiography

## Abstract

**Aims:**

The present study aimed to develop a comprehensive clinical- and echocardiography-based risk score for predicting cardiovascular (CV) adverse outcomes in patients with ischemic heart failure (IHF) and reduced left ventricular ejection fraction (LVEF).

**Methods:**

This retrospective cohort study included 1341 hospitalized patients with IHF and LVEF < 50% at our hospital from 2009 to 2017. Cox regression models and nomogram were utilized to develop a comprehensive prediction model (C&E risk score) for CV mortality and CV-related events (hospitalization or death).

**Results:**

Over a median 26-month follow-up, CV mortality and CV events rates were 17.4% and 40.9%, respectively. The C&E risk score, incorporating both clinical and echocardiographic factors, demonstrated superior predictive performance for CV outcomes compared to models using only clinical or echocardiographic factors. Internal validation confirmed the stable predictive ability of the C&E risk score, with an AUC of 0.740 (95% CI 0.709–0.775, *P* < 0.001) for CV mortality and an AUC of 0.678 (95% CI 0.642–0.696, *P* < 0.001) for CV events. Patients were categorized into low-, intermediate-, and high-risk based on the C&E risk score, with progressively increasing CV mortality (5.3% vs. 14.6% vs. 31.9%, *P* < 0.001) and CV events (28.8% vs. 38.2% vs. 55.0%, *P* < 0.001). External validation also confirmed the risk score’s prognostic efficacy within additional IHF patient datasets.

**Conclusion:**

This study establishes and validates the novel C&E risk score as a reliable tool for predicting CV outcomes in IHF patients with reduced LVEF. The risk score holds potential for enhancing risk stratification and guiding clinical decision-making for high-risk patients.

**Graphical abstract:**

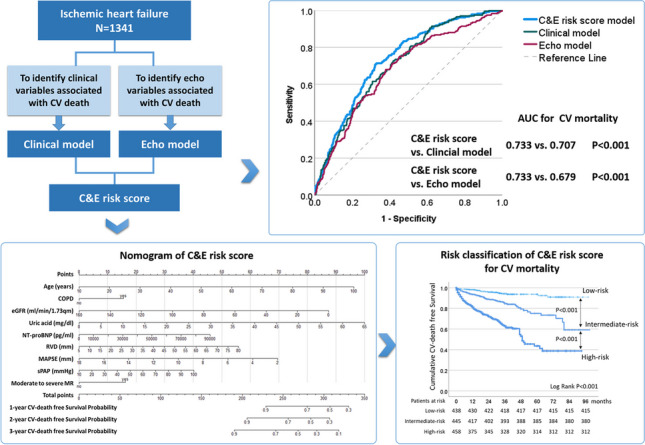

**Supplementary information:**

The online version contains supplementary material available at 10.1007/s00392-024-02399-1.

## Introduction

The prevalence of heart failure (HF) rises with the aging population and improved survival rates among patients with heart diseases due to modern treatment innovation [1]. Ischemic heart disease plays a significant role in contributing to HF and holds crucial prognostic implications across the spectrum of this condition [2]. Prognostic assessment is of importance for risk stratification and optimizing patient care [3, 4]. To achieve this, ongoing efforts focus on developing prognostic risk scores with satisfactory clinical performance for HF patients [5]. In patients with ischemic HF (IHF), demographic, clinical, and hemodynamic factors collectively influence outcomes [6–8]. Previous studies have identified that left ventricular function, coronary stenosis distribution, and severity were pivotal survival determinants in patients with stable angina [9]. Additionally, distinct risk factors such as diabetes, prior myocardial infarction, hypertension, and male gender have surfaced as contributors to cardiovascular (CV) mortality or myocardial infarction in individuals with stable angina [7].

Transthoracic echocardiography (TTE) is crucial for evaluating cardiac function in daily clinical practice, providing comprehensive insights encompassing chamber dimensions, ventricular hypertrophy, regional wall movement anomalies, right-side performance, valvular function, systolic and diastolic function [10–12]. Several clinical studies emphasize the prognostic significance of echocardiographic parameters in HF patients with reduced LVEF, suggesting that utilizing a single echocardiographic prognostic marker or a combination of multiple markers could be a valuable approach for prognostic stratification [13]. In addition to conventional echocardiographic metrics, advanced technology-driven parameters, particularly speckle tracking derived global longitudinal strain (GLS), have shown supplementary prognostic potential, sometimes surpassing the predictive performance of LVEF in chronic systolic HF [14]. Despite these insights, the incremental role of jointly assessed clinical indexes and cardiac imaging-derived parameters, especially echocardiographic parameters, remains less explored in the prognostic framework for IHF patients with reduced LVEF [6]. To address this knowledge gap, the present study aimed to identify independent clinical and echocardiographic parameters, including both standard echocardiography metrics and GLS, for predicting major adverse cardiovascular outcomes in IHF patients with LVEF < 50%. Our objective involves developing and validating a comprehensive clinical- and echocardiography-based risk score (C&E risk score) tailored for the risk stratification of IHF patients with LVEF < 50%.

## Methods

### Study population

**Fig. 1:**
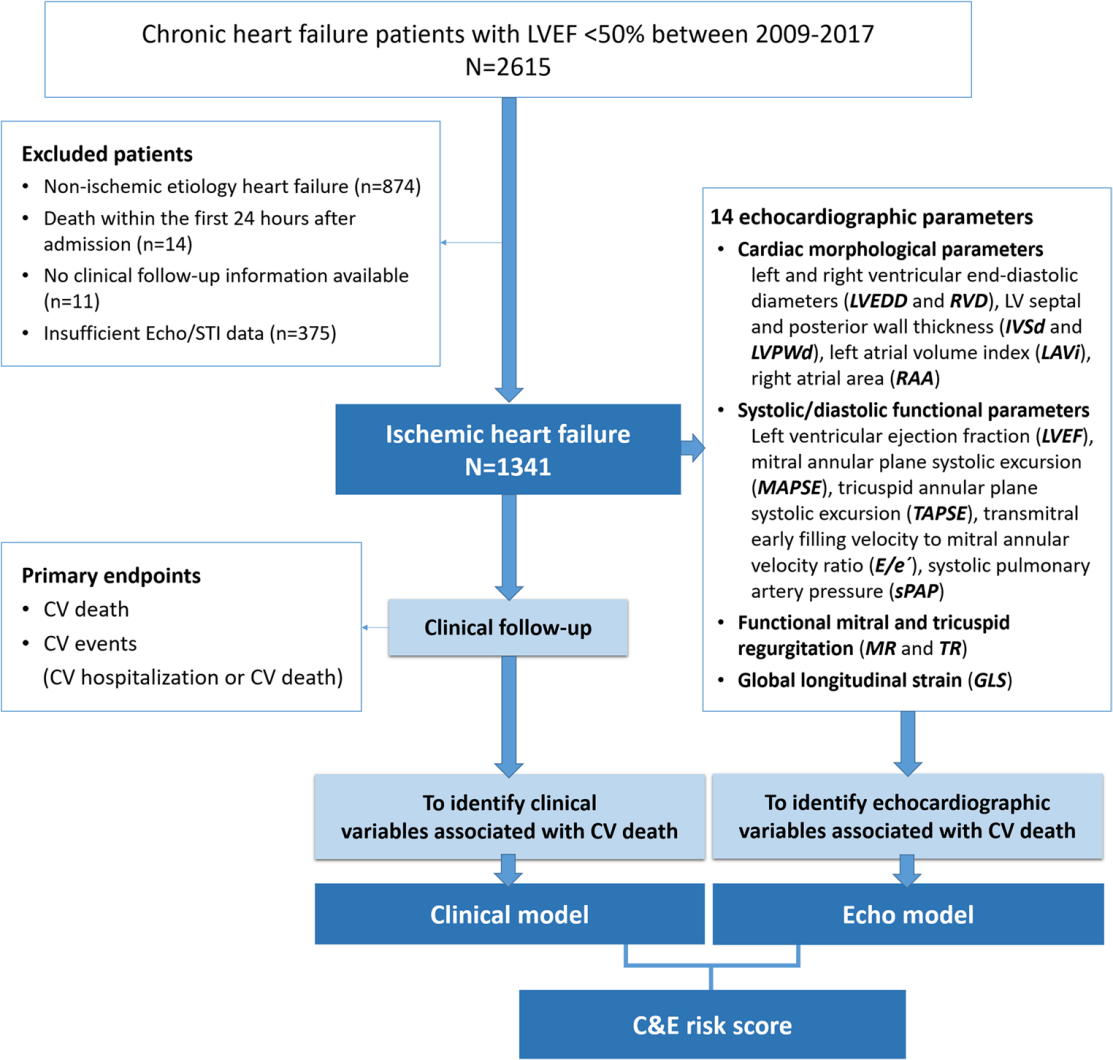
Study flowchart. CV, cardiovascular; Echo, echocardiography; GLS, global longitudinal strain; IVSd, end‐diastolic interventricular septal thickness; LAVi, left atrial volume indexed to body surface area; LVEDD, left ventricular end‐diastolic dimension; LVEF, left ventricular ejection fraction; LVPWd, end‐diastolic posterior wall thickness; MAPSE, mitral annular plane systolic excursion; MR, mitral regurgitation; RAA, end‐systolic right atrial area; RVD, end‐diastolic mid-right ventricular diameter; sPAP, systolic pulmonary artery pressure; STI, speckle tracking imaging; TAPSE, tricuspid annular plane systolic excursion; TR, tricuspid regurgitation

This retrospective cohort study comprised 1341 chronic HF patients with angiography-diagnosed ischemic heart disease and left ventricular systolic dysfunction (LVEF < 50%) admitted to our cardiology department from 2009 to 2017. Chronic HF diagnosis followed the current European Society of Cardiology guidelines [15]. Ischemic heart disease was confirmed clinically at baseline visit by coronary angiography defined stenosis of > 50% in ≥ 1 epicardial coronary artery with a visual reference lumen diameter of ≥ 2.5 mm, or patients had a history of myocardial infarction (MI), percutaneous coronary intervention (PCI), or coronary artery bypass graft (CABG) surgery [16]. De novo acute HF, malignancy, and other non-cardiac conditions limiting life expectancy to less than 1 year were excluded. HF patients with non-ischemic etiologies, including idiopathic dilated cardiomyopathy, valve heart disease, hypertensive heart disease, arrhythmias, conduction disturbances, chemotherapy-related cardiac dysfunction, myocarditis, infiltrative cardiomyopathy, hypertrophic cardiomyopathy, and other miscellaneous causes, were excluded. Figure 1 illustrates the study’s flowchart for developing a new prediction model.

### Ethics

The study was approved by the local Ethics Committee at the University of Würzburg and conducted in accordance to the Declaration of Helsinki. Written informed consent was obtained from patients and healthy volunteers.

### Echocardiographic measures

A comprehensive TTE examination was performed according to the American Society of Echocardiography (ASE) recommendations (Vivid 7 or IE9, GE Vingmed Ultrasound, Horten, Norway) [17, 18]. Standard measurements were performed offline using the dedicated software (EchoPAC™, version 202, GE Vingmed Ultrasound AS, Horten, Norway). Thirteen standard echocardiography parameters together with GLS were initially evaluated. LV end-diastolic dimension (LVEDD), end-diastolic thickness of the posterior wall (LVPWd), and the septum (IVSd) were measured using M-mode in the parasternal LV long axis view. Right ventricular end-diastolic mid dimension (RVD) and end-systolic right atrial area (RAA) were measured in the RV-focused apical 4-chamber view. Left atrial volume (LAV) was measured in the LV-focused apical 4-chamber view at end systole. LAVi was calculated by dividing LAV by body surface area of subjects. LVEF was measured by using the Simpson biplane method from the apical 2- and 4-chamber view. Septal and lateral mitral annular plane systolic excursion (MAPSE) were respectively measured with a cursor respectively placed on the septal and lateral side of the mitral annulus from the LV-focused apical 4‐chamber view by M‐mode imaging. Average MAPSE value at septal and lateral annulus was calculated. Tricuspid annular plane systolic excursion (TAPSE) was measured in the RV-focused apical 4-chamber view by M-mode imaging. Pulsed-wave Doppler-derived mitral peak velocity of early (E) and atrial (A) diastolic filling was measured. Tissue-Doppler-derived early diastolic mitral annular velocity (e´) was acquired at the septal and lateral mitral annular sites and then septal, lateral, and average E/e´ ratio were calculated. Peak tricuspid regurgitation jet velocity (TRV_max_) was measured with colour Doppler and continuous-wave Doppler. Systolic pulmonary artery pressure (sPAP) was derived from using the simplified Bernoulli equation in combination with an estimated right atrial pressure (RAP): sPAP = 4V^2^ + RAP, where V indicates the TRV_max_. RAP was estimated through inferior vena cava diameter and its respiratory variation. Functional mitral regurgitation (MR) and tricuspid regurgitation (TR) were assessed, and their severity was graded as mild, moderate, or severe. Two-dimensional speckle tracking-derived longitudinal strain analysis was conducted offline in standard LV apical views (4-, 2-, and 3-chamber) with a frame rate ranging from 50 to 80 frames per second, spanning three consecutive cardiac cycles. This analysis was performed using the EchoPAC PC Software (version 202, GE Vingmed Ultrasound AS, Horten, Norway). Region of interest (ROI) was created by manually outlining the endocardial border on each LV apical view at the end-systolic frame. The system automatically tracked the tissue within the region and divided the myocardium into standard 18 LV segments. The trace analysis was automatically displayed after validating the tracking. GLS was automatically calculated by averaging the segmental strain of all 18 LV segments.

### Outcomes

In this study, we initially conducted a thorough review of hospitalization records for each patient throughout the follow-up period. The primary reasons for hospitalization or death, along with the corresponding dates, were extracted. For patients without documented endpoints in hospital records, outcomes were assessed through telephone interviews with the patients or their family members. The outcome was further confirmed by contacting their general practitioners for detailed information. The primary endpoints were CV death and combined CV events included CV-related hospitalization or CV death. CV deaths were defined as deaths that result from an acute MI, sudden cardiac death, death due to HF, death due to CV procedures, death due to CV hemorrhage, death due to stroke, pulmonary embolism, peripheral arterial disease, or heart transplantation [19]. The CV causes of hospitalization included HF, acute/chronic coronary syndrome, uncontrolled hypertension, arrhythmia/atrial fibrillation, worsening renal function/acute renal dysfunction/cardiorenal syndrome, pulmonary embolism, or peripheral arterial disease.

### Statistical analysis

Statistical analyses were performed using SPSS 28.0 (SPSS Inc., Chicago, IL, USA) and R Statistical Software (version 4.3.0; R Core Team 2022) along with Storm Statistical Platform (www.medsta.cn/software). Two-sided *P* values less than 0.05 were considered statistically significant in each statistical analysis.

Continuous variables were presented as mean (standard deviation) or median (interquartile range, IQR). Normal distribution of all continuous variables was checked by inspecting Q–Q plots and Shapiro–Wilk test. Continuous variables were compared by unpaired Student’s test or Mann–Whitney *U*-test. Differences cross three or four groups were compared using Kruskal–Wallis H test. Categorical or dichotomous variables were expressed as count and percent, and the differences among groups were compared using chi-square test.

The development of the prediction model was based on Cox proportional hazard regression analysis. In detail, a multiple imputation procedure utilizing random once imputation (a maximum of 50 iterations) was employed to address missing values in the major study variables identified (< 10%) prior to the primary analyses. Potential predictive variables associated with CV mortality were then explored through inter-group comparisons and univariate Cox regression analysis. Subsequently, variables showing statistical significance (*P* values < 0.05) were included in multivariable Cox regression analysis. Independent variables significantly associated with CV mortality were identified for the establishment of the final prediction model by multivariable Cox regression analysis, which was achieved using a stepwise backward elimination process based on likelihood ratio. Hazard ratio (HR) and 95% confidence interval (CI) were estimated. The variance inflation factor (VIF) was computed to assess the presence of potential multicollinearity among continuous variables. Multicollinearity was considered significant when the VIF > 5. In cases of multicollinearity, only one variable with the highest Wald value derived from univariate Cox regression analysis was retained for analysis. The nomogram representing the final multivariable Cox regression model results was constructed to visualize the developed prediction model, denoted as the C&E risk score, using the Regression Modelling Strategies R package (v6.6–0; Frank Harrell 2023) and the normogramFormula package (v1.2.0.0; Jing Zhang, Zhi Jin). To evaluate the discrimination ability of the models, receiver operating characteristic (ROC) curves based on Harrell’s concordance index (C-index) were employed. The area under the ROC curves (AUC) and 95% CI was assessed. To thoroughly assess the improvement in the prediction model's performance, we employed the net reclassification improvement (NRI) and integrated discrimination index (IDI). Each patient was assigned a risk score based on the established nomogram. Using tertiles of their respective risk scores, patients were categorized into low-risk, intermediate-risk, and high-risk groups. The prognostic significance of these three risk groups was evaluated through survival curves, and statistical differences were assessed using the log-rank test.

The R statistical software was utilized for both internal and external validation of the developed prediction model. Internal validation was performed by a bootstrap resampling approach with 1000 replications to assess the stability of the prediction model within the development cohort (i.e., the training set), comprising hospitalized IHF patients from 2009 to 2017. To evaluate the generalizability of the developed prediction model, external validation was conducted in three distinct patient sets: validation set 1 (IHF patients hospitalized in the year of 2018, excluding those rehospitalized in our hospital), validation set 2 (IHF patients treated with angiotensin receptor/neprilysin inhibitor (ARNIs) therapy hospitalized between 2016 and 2018), and validation set 3 (a combined cohort of IHF and non-IHF patients hospitalized in the year of 2018, excluding those rehospitalized in our hospital). The ROC curves, based on the C-index for predicting CV mortality and CV events risk, were then compared between the three validation sets and the training set.

## Results

### Baseline clinical characteristics of IHF patients with HFmrEF and HFrEF

Among 1341 IHF patients with LVEF < 50%, 595 (44.4%) had HFmrEF (LVEF 41–49%), and 746 patients (55.6%) had HFrEF (LVEF ≤ 40%, Table 1). The median clinical follow-up was 26 months (IQR 14–39). A total of 376 patients (28.0%) died, and 5 underwent heart transplantation (0.5%). CV mortality and CV events occurred at rates of 17.4% and 40.9%, respectively, with higher rates in HFrEF than HFmrEF groups (CV mortality: 20.8% vs. 13.3%, *P* < 0.001; CV events: 44.6% vs. 36.1%, *P* = 0.002). In this IHF cohort, baseline characteristics included a mean age of 70 ± 11 years, 79.6% males, 33.8% with NYHA class III or IV symptoms, 60.9% with a history of MI, 54.9% with PCI, and 28.4% had received CABG surgery. HFrEF patients, compared to HFmrEF, showed higher prevalence of NYHA class III or IV, higher systolic blood pressure, a lower prevalence of PCI, a higher prevalence of atrial fibrillation, hyperuricemia, renal dysfunction, peripheral vascular disease, chronic obstructive pulmonary disease (COPD), sleep disorders, and implantable cardioverter defibrillator (ICD) or cardiac resynchronization therapy with defibrillator (CRT-D) implantation. Laboratory findings displayed differences between HFmrEF and HFrEF, including markers of renal function, uric acid, total cholesterol, triglycerides, and N-terminal pro–B-type natriuretic peptide (NT-proBNP). Echocardiography data showed median LVEF of 39.0% (32.0–45.0)%, GLS of 9.9% (7.7–12.4)%, and significant differences in standard echocardiographic measures, including GLS, between the two groups.

**Table 1:** Clinical and echocardiographic characteristics in ischemic heart disease patients with HFmrEF and HFrEF

	Total	HFmrEF LVEF 41%—49%	HFrEF LVEF ≤ 40%	*P* value
No	1341 (100)	595 (44.4)	746 (55.6)	
Age (years)	70 ± 11	70 ± 12	71 ± 11	0.069
Male [n (%)]	1067 (79.6)	462 (77.6)	605 (81.1)	0.119
Body mass index [kg/m^2^]	27.6 ± 4.6	27.8 ± 4.8	27.4 ± 4.5	0.068
NYHA class [n (%)]				< 0.001
I	405 (30.2)	221 (37.1)	184 (24.7)*	
II	483 (36.0)	221 (37.1)	262 (35.1)	
III	357 (26.6)	128 (21.5)	229 (30.7)*	
IV	96 (7.2)	25 (4.2)	71 (9.5)*	
Systolic blood pressure (mmHg)	129 ± 24	133 ± 24	126 ± 23	< 0.001
Diastolic blood pressure (mmHg)	73 ± 14	73 ± 14	73 ± 14	0.879
Angina pectoris [n (%)]	407 (30.4)	176 (29.6)	231 (31.0)	0.584
Prior myocardial infarction [n (%)]	816 (60.9)	374 (62.9)	442 (59.2)	0.179
PCI [n (%)]	736 (54.9)	352 (59.2)	384 (51.5)	0.005
CABG [n (%)]	381 (28.4)	165 (27.7)	216 (29.0)	0.622
Comorbidities [n (%)]				
Atrial fibrillation	386 (28.8)	147 (24.7)	239 (32.0)	0.003
Obesity	516 (38.5)	242 (40.7)	274 (36.7)	0.140
Hypertension	994 (74.1)	447 (75.1)	547 (73.3)	0.454
Diabetes	478 (35.6)	196 (32.9)	282 (37.8)	0.065
Hyperlipidemia	550 (41.0)	239 (40.2)	311 (41.7)	0.574
Smoking status				0.237
Never smoked	850 (63.4)	384 (64.5)	466 (62.5)	
Ex-smoking	252 (18.8)	100 (16.8)	152 (20.4)	
Currently smoking	239 (17.8)	111 (18.7)	128 (17.2)	
Hyperuricemia	555 (41.4)	197 (33.1)	358 (48.0)	< 0.001
Anemia	805 (60.0)	358 (60.2)	447 (59.9)	0.926
Renal dysfunction	590 (44.0)	234 (39.3)	356 (47.4)	0.002
Stroke / TIA	133 (9.9)	50 (8.4)	83 (11.1)	0.097
Peripheral vascular disease	144 (10.7)	49 (8.2)	95 (12.7)	0.008
COPD	179 (13.3)	62 (10.4)	117 (15.7)	0.005
Sleep disorders	83 (6.2)	28 (4.7)	55 (7.4)	0.044
ICD / CRT-D implantation [n (%)]	245 (18.3)	56 (9.4)	189 (25.3)	< 0.001
Laboratory data				
eGFR (ml/min/1.73qm)	64 (47–81)	67 (49–83)	61 (45–80)	0.003
Creatinine (mg/dl)	1.13 (0.93–1.49)	1.09 (0.91–1.40)	1.18 (0.95–1.51)	< 0.001
Urea (mg/dl)	43.1 (31.5–61.0)	38.5 (29.2–55.8)	46.3 (34.0–66.4)	< 0.001
C-reactive protein (mg/dl)	1.01 (0.32–3.05)	1.05 (0.28–3.32)	0.99 (0.36–2.74)	0.963
Uric acid (mg/dl)	6.7 (5.3–8.4)	6.3 (5.1–7.7)	7.1 (5.6–8.8)	< 0.001
Hemoglobin (g/dl)	13.0 (11.3–14.3)	13.0 (11.4–14.2)	13.0 (11.3–14.4)	0.430
Total cholesterol (mg/dl)	161 (135–191)	163 (138–196)	159 (133–189)	0.016
Triglyceride (mg/dl)	123 (94–169)	125 (97–181)	120 (90–163)	0.003
HDLC (mg/dl)	42 (33–53)	43 (34–53)	41 (32–52)	0.086
LDLC (mg/dl)	89 (69–114)	91 (69–116)	89 (68–112)	0.187
NT-proBNP (pg/ml)	2412 (904–7085)	1930 (662–4877)	3169 (1140–8319)	< 0.001
hsTnT (pg/ml)	72.1 (276.0–536.5) *n* = 555	98.2 (26.4–993.0) *n* = 263	60.1 (27.9–227.2) *n* = 292	0.026
Medications [n (%)]				
Beta-blockers	1142 (85.2)	506 (85.0)	636 (85.3)	0.913
ACEIs / ARBs	1099 (82.0)	495 (83.2)	604 (81.0)	0.292
MRAs	422 (31.5)	122 (20.5)	300 (40.2)	< 0.001
Loop diuretics	779 (58.1)	285 (47.9)	494 (66.2)	< 0.001
Digoxin	153 (11.4)	48 (8.1)	105 (14.1)	< 0.001
^a^ARNIs	107 (8.0)	22 (3.7)	85 (11.4)	< 0.001
Echocardiography				
Cardiac morphology				
LVEDD (mm)	55.0 (50.0–60.0)	52.0 (48.0–57.0)	57.0 (52.0–63.0)	< 0.001
IVSd (mm)	10.0 (8.0–11.0)	10.0 (9.0–11.0)	10.0 (8.0–11.0)	< 0.001
LVPWd (mm)	9.0 (8.0–11.0)	10.0 (9.0–11.0)	9.0 (8.0–10.0)	< 0.001
IVSd or LVPWd ≥ 11 mm [n (%)]	537 (40.0)	277 (46.6)	260 (34.9)	< 0.001
LAVi (ml/m^2^)	39.2 (28.1–50.7)	36.8 (25.4–47.7)	41.3 (31.4–53.2)	< 0.001
RVD (mm)	28.0 (22.0–33.0)	28.0 (22.0–32.0)	29.0 (23.0–34.0)	0.003
RAA (cm^2^)	17.5 (14.0–22.0)	17.0 (13.3–21.0)	18.0 (14.2–23.0)	< 0.001
Cardiac function				
LVEF (%)	39.0 (32.0–45.0)	46.0 (43.0–48.0)	32.0 (26.0–37.0)	< 0.001
MAPSE(mm)	8.0 (6.5–10.0)	9.0 (7.5–10.5)	7.5 (6.0–8.5)	< 0.001
TAPSE (mm)	17.0 (13.0–21.0)	18.2 (15.0–22.0)	15.0 (12.0–19.5)	< 0.001
E/e´ ratio	14.5 (10.6–20.0)	12.7 (9.3–16.7)	15.9 (11.8–22.0)	< 0.001
sPAP (mmHg)	34.0 (26.0–46.0)	32.0 (24.0–41.0)	37.0 (27.0–49.0)	< 0.001
Moderate to severe MR [n (%)]	214 (16.0)	72 (12.1)	142 (19.0)	< 0.001
Moderate to severe TR [n (%)]	106 (7.9)	35 (5.9)	71 (9.5)	0.014
GLS (%)	9.9 (7.7–12.4)	11.9 (10.0–14.0)	8.2 (6.2–10.2)	< 0.001
Clinical outcomes				
Follow-up duration (months)	26 (14–39)	27 (16–39)	24 (12–39)	0.009
Alive [n (%)]	960 (71.6)	464 (78.0)	496 (66.5)	< 0.001
Death [n (%)]	376 (28.0)	130 (21.8)	246 (33.0)	
Heart transplantation [n (%)]	5 (0.4)	1 (0.2)	4 (0.5)	
CV mortality [n (%)]	234 (17.4)	79 (13.3)	155 (20.8)	< 0.001
CV hospitalization [n (%)]	424 (31.6)	174 (29.2)	250 (33.5)	0.095
Combined CV events [n (%)]	548 (40.9)	215 (36.1)	333 (44.6)	0.002

Data are expressed as mean ± standard deviation, median with interquartile range (Q1-Q3), or as number (%)

*P < 0.05 vs. HFmrEF group. ^a^ARNIs therapy began in 2016

ACEIs, angiotensin-converting enzyme inhibitors; ARBs, angiotensin II receptor antagonists; ARNIs, angiotensin receptor/neprilysin inhibitors; CABG, coronary artery bypass grafting; COPD, chronic obstructive pulmonary disease; CRT-D, cardiac resynchronization therapy with defibrillator; CV, cardiovascular; E/e´ ratio, the ratio of early diastolic mitral inflow velocity to mitral annular tissue velocity; eGFR, estimated glomerular filtration rate; GLS, global longitudinal strain; HDLC, high-density lipoprotein cholesterol; HFmrEF, heart failure with mildly reduced ejection fraction; HFrEF, heart failure with reduced ejection fraction; hsTnT, high-sensitive troponin T; ICD, implantable cardioverter defibrillator; IVSd, end‐diastolic interventricular septal thickness; LAVi, left atrial volume indexed to body surface area; LDLC, low-density lipoprotein cholesterol; LVEDD, left ventricular end‐diastolic dimension; LVEF, left ventricular ejection fraction; LVPWd, end‐diastolic posterior wall thickness; MAPSE, mitral annular plane systolic excursion; MR, mitral regurgitation; MRAs, mineralocorticoid receptor antagonists; NT-proBNP, N-terminal prohormone of brain natriuretic peptide; NYHA, New York Heart Association; PCI, percutaneous coronary intervention; RAA, end‐systolic right atrial area; RVD, end‐diastolic mid‐right ventricular diameter; sPAP, systolic pulmonary artery pressure; TAPSE, tricuspid annular plane systolic excursion; TIA, transient ischemic attack; TR, tricuspid regurgitation

### Development of a comprehensive prediction model for CV mortality — C&E risk score

As shown in Table 2, initial assessment of clinical factors linked to CV mortality included age, NYHA class, diastolic blood pressure, PCI, atrial fibrillation, hyperuricemia, anemia, renal dysfunction, peripheral vascular disease, COPD, eGFR, creatinine, urea, C-reactive protein, uric acid, hemoglobin, total cholesterol, and NT-proBNP. These factors were identified based on a *P* value < 0.05 in inter-group comparisons between the no CV-death and CV-death groups, as well as in the univariate Cox regression model. Similarly, employing the same method, we identified, among the 14 observed echocardiographic parameters, that LAVi, RVD, RAA, LVEF, MAPSE, TAPSE, E/e´ ratio, sPAP, moderate to severe MR or TR, and GLS were significantly associated with CV mortality.

**Table 2:** Clinical and echocardiographic characteristics in ischemic heart failure patients with and without CV death

	No CV-death	CV death	*P* value	Univariate HR (95% CI)	*P* value
No	1107 (82.6)	234 (17.4)			
Age (years)	69 ± 12	74 ± 10	< 0.001	1.047 (1.034–1.061)	< 0.001
Male [n (%)]	881 (79.6)	186 (79.5)	0.973		
Body mass index [kg/m^2^]	27.6 ± 4.6	27.2 ± 4.6	0.083		
NYHA class [n (%)]			< 0.001		
I	366 (33.1)	39 (16.7)*		reference	
II	396 (35.8)	87 (37.2)		1.760 (1.206–2.568)	0.003
III	281 (25.4)	76 (32.5)*		2.529 (1.717–3.724)	< 0.001
IV	64 (5.8)	32 (13.7)*		3.177 (1.986–5.081)	< 0.001
Systolic blood pressure (mmHg)	130 ± 24	128 ± 25	0.087		
Diastolic blood pressure (mmHg)	74 ± 14	71 ± 15	0.002	0.985 (0.976–0.995)	0.002
Angina pectoris [n (%)]	341 (30.8)	66 (28.2)	0.432		
Prior myocardial infarction [n (%)]	672 (60.7)	144 (61.5)	0.812		
PCI [n (%)]	625 (56.5)	111 (47.4)	0.012	0.693 (0.536–0.896)	0.005
CABG [n (%)]	305 (27.6)	76 (32.5)	0.129		
Comorbidities [n (%)]					
Atrial fibrillation	298 (26.9)	88 (37.6)	0.001	1.568 (1.204–2.044)	< 0.001
Obesity	436 (39.4)	80 (34.2)	0.138		
Hypertension	817 (73.8)	177 (75.6)	0.560		
Diabetes	382 (34.5)	96 (41.0)	0.059		
Hyperlipidemia	466 (42.1)	84 (35.9)	0.080		
Smoking status			0.073		
Never smoked	696 (62.9)	154 (65.8)			
Ex-smoking	202 (18.2)	50 (21.4)			
Currently smoking	209 (18.9)	30 (12.8)*			
Hyperuricemia	414 (37.4)	141 (60.3)	< 0.001	2.333 (1.795–3.031)	< 0.001
Anemia	647 (58.4)	158 (67.5)	0.010	1.599 (1.215–2.104)	< 0.001
Renal dysfunction	437 (39.5)	153 (65.4)	< 0.001	2.975 (2.269–3.899)	< 0.001
Stroke / TIA	104 (9.4)	29 (12.4)	0.163		
Peripheral vascular disease	108 (9.8)	36 (15.4)	0.012	1.728 (1.211–2.465)	0.003
COPD	134 (12.1)	45 (19.2)	0.004	1.719 (1.241–2.380)	0.001
Sleep disorders	62 (5.6)	21 (9.0)	0.052		
ICD / CRT-D implantation	194 (17.5)	51 (21.8)	0.125		
Laboratory data					
eGFR (ml/min/1.73qm)	68 (49–84)	52 (35–68)	< 0.001	0.976 (0.971–0.982)	< 0.001
Creatinine (mg/dl)	1.10 (0.91–1.41)	1.37 (1.10–1.85)	< 0.001	1.222 (1.149–1.299)	< 0.001
Urea (mg/dl)	40.7 (30.3–57.5)	56.0 (39.5–83.8)	< 0.001	1.013 (1.010–1.015)	< 0.001
C-reactive protein (mg/dl)	0.93 (0.30–2.97)	1.43 (0.44–3.49)	0.005	^a^1.156 (1.065–1.255)	< 0.001
Uric acid (mg/dl)	6.5 (5.3–8.0)	7.7 (5.8–9.7)	< 0.001	1.054 (1.035–1.073)	< 0.001
Hemoglobin (g/dl)	13.1 (11.5–14.4)	12.4 (10.8–14.0)	< 0.001	0.871 (0.822–0.923)	< 0.001
Total cholesterol (mg/dl)	162 (137–192)	156 (129–187)	0.023	0.995 (0.992–0.998)	< 0.001
Triglyceride (mg/dl)	124 (95–169)	114 (90–167)	0.090		
HDLC (mg/dl)	42 (34–53)	39 (31–50)	0.015	0.992 (0.983–1.001)	0.069
LDLC (mg/dl)	90 (69–114)	88 (68–112)	0.327		
NT-proBNP (pg/ml)	2061 (703–5810)	4700 (1800–14451)	< 0.001	^a^1.527 (1.393–1.673)	< 0.001
hsTnT (pg/ml)	67.7 (26.3–560.5) *n* = 471	81.6 (43.0–401.3) *n* = 292	0.261		
Medications [n (%)]					
Beta-blockers	947 (85.5)	195 (83.3)	0.387		
ACEIs / ARBs	928 (83.8)	171 (73.1)	< 0.001	0.511 (0.382–0.682)	< 0.001
MRAs	350 (31.6)	72 (30.8)	0.800		
Loop diuretics	599 (54.1)	180 (76.9)	< 0.001	2.856 (2.106–3.873)	< 0.001
Digoxin	99 (8.9)	54 (23.1)	< 0.001		
^ b^ARNIs	97 (8.8)	10 (4.3)	0.021	0.500 (0.265–0.942)	0.032
Echocardiography					
Cardiac morphology					
LVEDD (mm)	55.0 (50.0–60.0)	56.0 (50.0–62.1)	0.067		
IVSd (mm)	10.0 (8.0–11.0)	10.0 (9.0–11.0)	0.531		
LVPWd (mm)	9.0 (8.0–10.7)	10.0 (8.0–11.0)	0.094		
IVSd or LVPWd ≥ 11 mm [n (%)]	439 (39.7)	98 (41.9)	0.528		
LAVi (ml/m^2^)	38.0 (27.3–49.8)	44.9 (32.9–54.6)	< 0.001	1.010 (1.006–1.014)	< 0.001
RVD (mm)	28.0 (22.0–33.0)	30.0 (24.0–36.0)	< 0.001	1.037 (1.021–1.053)	< 0.001
RAA (cm^2^)	17.0 (14.0–21.8)	19.0 (15.0–25.0)	< 0.001	1.040 (1.024–1.057)	< 0.001
Cardiac function					
LVEF (%)	40.0 (32.0–45.0)	36.0 (28.0–43.0)	< 0.001	0.966 (0.953–0.979)	< 0.001
MAPSE(mm)	8.0 (6.5–10.0)	7.0 (6.0–8.8)	< 0.001	0.826 (0.779–0.875)	< 0.001
TAPSE (mm)	17.0 (13.0–21.0)	15.0 (12.0–19.0)	< 0.001	0.936 (0.912–0.960)	< 0.001
E/e´ ratio	13.8 (10.0–19.6)	17.0 (12.9–23.7)	< 0.001	1.040 (1.027–1.053)	< 0.001
sPAP (mmHg)	33.0 (25.0–43.0)	42.0 (30.0–54.0)	< 0.001	1.033 (1.025–1.041)	< 0.001
Moderate to severe MR [n (%)]	147 (13.3)	67 (28.6)	< 0.001	2.539 (1.911–3.372)	< 0.001
Moderate to severe TR [n (%)]	73 (6.6)	33 (14.1)	< 0.001	2.379 (1.645–3.440)	< 0.001
GLS (%)	10.2 (7.9–12.5)	8.8 (6.4–10.9)	< 0.001	0.892 (0.857–0.927)	< 0.001

Data are expressed as mean ± standard deviation, median with interquartile range (Q1-Q3), or as number (%)

*P < 0.05 vs. no CV-death group

^a^natural logarithm (Ln) transformation; ^b^ARNIs therapy began in 2016

ACEIs, angiotensin-converting enzyme inhibitors; ARBs, angiotensin II receptor antagonists; ARNIs, angiotensin receptor/neprilysin inhibitors; CABG, coronary artery bypass grafting; CI, confidence interval; COPD, chronic obstructive pulmonary disease; CRT-D, cardiac resynchronization therapy with defibrillator; CV, cardiovascular; E/e´ ratio, the ratio of early diastolic mitral inflow velocity to mitral annular tissue velocity; eGFR, estimated glomerular filtration rate; GLS, global longitudinal strain; HDLC, high-density lipoprotein cholesterol; HR, hazard ratio; hsTnT, high-sensitive troponin T; ICD, implantable cardioverter defibrillator; IVSd, end‐diastolic interventricular septal thickness; LAVi, left atrial volume indexed to body surface area; LDLC, low-density lipoprotein cholesterol; LVEDD, left ventricular end‐diastolic dimension; LVEF, left ventricular ejection fraction; LVPWd, end‐diastolic posterior wall thickness; MAPSE, mitral annular plane systolic excursion; MR, mitral regurgitation; MRAs, mineralocorticoid receptor antagonists; NT-proBNP, N-terminal prohormone of brain natriuretic peptide; NYHA, New York Heart Association; PCI, percutaneous coronary intervention; RAA, end‐systolic right atrial area; RVD, end‐diastolic mid‐right ventricular diameter; sPAP, systolic pulmonary artery pressure; TAPSE, tricuspid annular plane systolic excursion; TIA, transient ischemic attack; TR, tricuspid regurgitation

Multivariable Cox regression analysis with a stepwise backward elimination process, presented in Table 3, revealed that older age, COPD, lower eGFR and total cholesterol levels, higher uric acid levels, and Ln-transformed NT-proBNP were independent risk factors of CV mortality, and these variables were thus integrated into the clinical model. The echocardiographic independent risk factors eligible for the model included RVD, MAPSE, E/e´ ratio, sPAP, moderate to severe MR, and GLS. Subsequently, a comprehensive prediction model termed the C&E risk score was tested by integrating both the clinical and echocardiographic variables. Total cholesterol, E/e´ ratio, and GLS were excluded through the 4-step backward elimination process based on likelihood ratio. The final C&E risk score includes the following variables: age (HR 1.024, 95% CI 1.010–1.038, *P* = 0.001), COPD (HR 1.445, 95% CI 1.040–2.007, *P* = 0.028), eGFR (HR 0.989, 95% CI 0.983–0.995, *P* < 0.001), uric acid (HR 1.037, 95% CI 1.007–1.067, *P* = 0.014), NT-proBNP (HR 1.231, 95% CI 1.103–1.374, *P* < 0.001), RVD (HR 1.017, 95% CI 1.001–1.033, *P* = 0.041), MAPSE (HR 0.909, 95% CI 0.855–0.966, *P* = 0.002), sPAP (HR 1.010, 95% CI 1.000–1.019, *P* = 0.052), and moderate to severe MR (HR 1.432, 95% CI 1.052–1.949, *P* = 0.023).

**Table 3:** Development of the C&E risk score by employing multivariable Cox proportional hazards regression models based on clinical and echocardiographic parameters for predicting CV mortality in patients with IHF

	Multivariable models for CV mortality by employing a stepwise backward elimination process based on likelihood-ratio			
Clinical model: based on clinical variables				
	Step 1 (initial model)		Step 8 (final model)	
	HR (95% CI)	*P* value	HR (95% CI)	*P* value
Age (years)	1.026 (1.012–1.041)	< 0.001	1.029 (1.015–1.043)	< 0.001
DBP (mmHg)	0.993 (0.983–1.002)	0.146	-	-
NYHA class III-IV	1.194 (0.912–1.564)	0.196	-	-
PCI	0.856 (0.659–1.112)	0.224	-	-
Atrial fibrillation	1.128 (0.857–1.485)	0.389	-	-
Peripheral vascular disease	1.296 (0.901–1.863)	0.162	-	-
COPD	1.350 (0.969–1.881)	0.077	1.416 (1.018–1.970)	0.039
eGFR (ml/min/1.73qm)	0.989 (0.983–0.996)	0.001	0.989 (0.982–0.995)	< 0.001
^a^Ln (C-reactive protein)	1.045 (0.950–1.150)	0.367	-	-
Uric acid (mg/dl)	1.047 (1.020–1.076)	< 0.001	1.051 (1.024–1.078)	< 0.001
Hemoglobin (g/dl)	1.016 (0.947–1.090)	0.657	-	-
Total cholesterol (mg/dl)	0.997 (0.994–1.000)	0.083	0.997 (0.994–0.999)	0.020
^a^Ln (NT-proBNP)	1.250 (1.116–1.400)	< 0.001	1.280 (1.148–1.426)	< 0.001
Echo model: based on echocardiographic variables				
	Step 1 (initial model)		Step 6 (final model)	
	HR (95% CI)	*P* value	HR (95% CI)	*P* value
LAVi (ml/m^2^)	1.002 (0.996–1.009)	0.477	-	-
RVD (mm)	1.016 (0.997–1.035)	0.093	1.018 (1.001–1.035)	0.038
RAA (cm^2^)	0.994 (0.971–1.018)	0.641	-	-
LVEF (%)	0.997 (0.978–1.016)	0.773	-	-
MAPSE (mm)	0.933 (0.866–1.004)	0.065	0.920 (0.857–0.988)	0.021
TAPSE (mm)	0.985 (0.955–1.016)	0.336	-	-
E/e´ ratio	1.012 (0.997–1.027)	0.114	1.014 (0.999–1.029)	0.073
sPAP (mmHg)	1.016 (1.006–1.026)	0.001	1.017 (1.007–1.026)	< 0.001
Moderate to severe MR	1.604 (1.169–2.201)	0.003	1.666 (1.225–2.267)	0.001
Moderate to severe TR	1.167 (0.774–1.761)	0.461	-	-
GLS (%)	0.966 (0.912–1.023)	0.241	0.959 (0.915–1.005)	0.081
Combined model (C&E risk score): based on clinical and echocardiographic variables				
	Step 1 (initial model)		Step 4 (final model)	
	HR (95% CI)	*P* value	HR (95% CI)	*P* value
Age (years)	1.026 (1.011–1.041)	< 0.001	1.024 (1.010–1.038)	0.001
COPD	1.414 (1.017–1.964)	0.039	1.445 (1.040–2.007)	0.028
eGFR (ml/min/1.73qm)	0.989 (0.982–0.995)	< 0.001	0.989 (0.983–0.995)	< 0.001
Uric acid (mg/dl)	1.037 (1.005–1.071)	0.025	1.037 (1.007–1.067)	0.014
Total cholesterol (mg/dl)	0.998 (0.996–1.001)	0.291	-	-
^a^Ln (NT-proBNP)	1.208 (1.081–1.351)	< 0.001	1.231 (1.103–1.374)	< 0.001
RVD (mm)	1.015 (0.999–1.032)	0.075	1.017 (1.001–1.033)	0.041
MAPSE (mm)	0.939 (0.875–1.009)	0.085	0.909 (0.855–0.966)	0.002
E/e´ ratio	1.008 (0.993–1.024)	0.296	-	-
sPAP (mmHg)	1.007 (0.997–1.017)	0.148	1.010 (1.000–1.019)	0.052
Moderate to severe MR	1.452 (1.064–1.982)	0.019	1.432 (1.052–1.949)	0.023
GLS (%)	0.969 (0.925–1.015)	0.184	-	-

^a^natural logarithm (Ln) transformation

Abbreviations as in the Table 1

### Discrimination ability of the C&E risk score

**Fig. 2:**
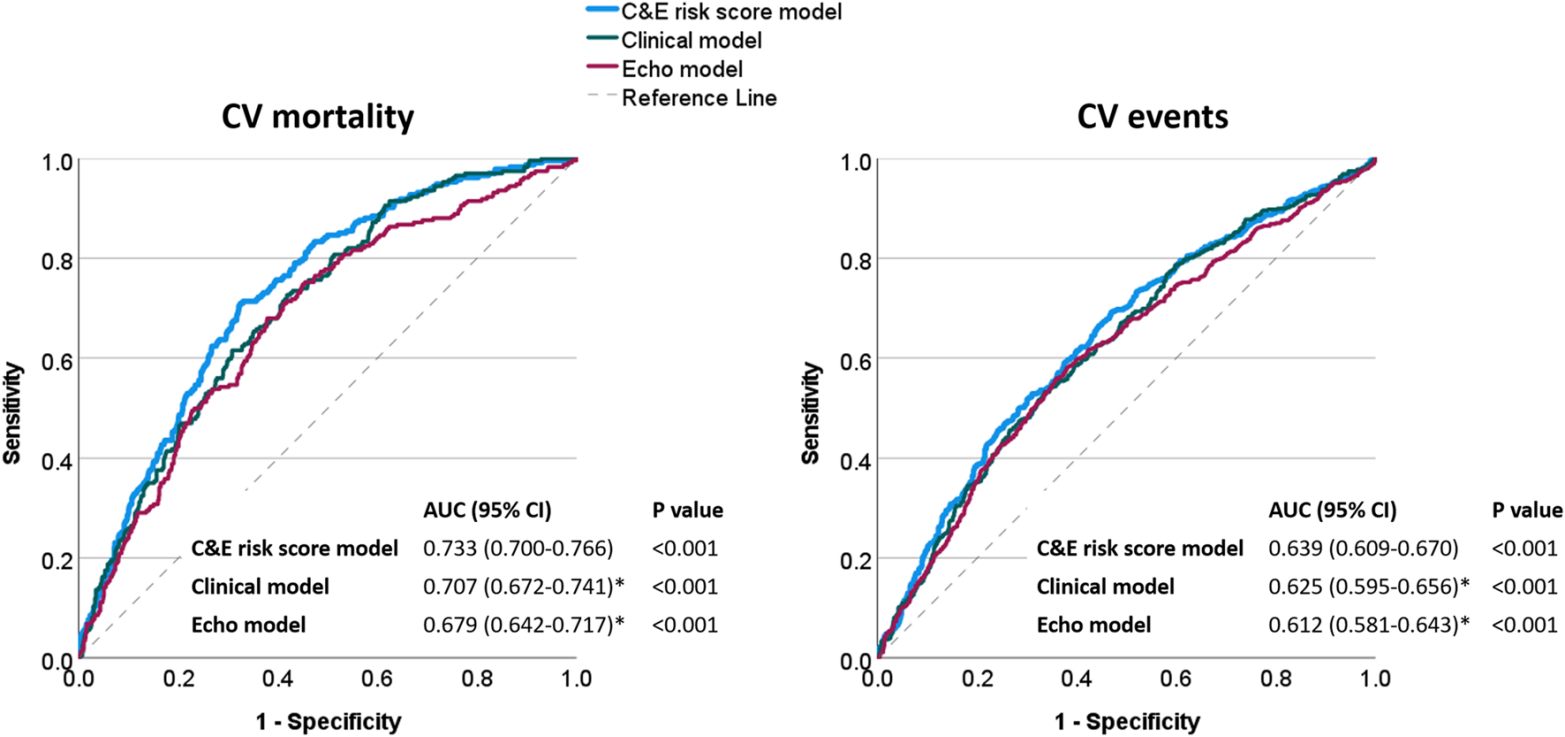
Comparison of discrimination abilities of the clinical model, the echocardiographic model, and the combined model (C&E risk score) using ROC curves based on Harrell’s concordance index (C-index). Notably, the combined prediction model (C&E risk score) demonstrates significantly enhanced discriminative capability for predicting CV mortality and CV events risk compared to the clinical or echocardiographic model in isolation. * *P* < 0.05 vs. C&E risk score model. AUC, area under the curve; CI, confidence interval; CV, cardiovascular; IHF, ischemic heart failure; ROC, receiver operating characteristic

The ROC curves based on the C-index underscored the discrimination ability of our prediction models (Fig. 2). The C&E risk score exhibited meaningful discriminatory power for CV mortality, with an AUC of 0.733 (95% CI 0.700–0.766, *P* < 0.001). The sensitivity was 0.709 (0.646–0.766), specificity was 0.675 (0.647–0.703), positive predictive value (PPV) was 0.316 (0.277–0.358), and negative predictive value (NPV) was 0.917 (0.895–0.934). Additionally, the C&E risk score demonstrated modest discriminatory performance for CV events, yielding an AUC of 0.639 (0.609–0.670, *P* < 0.001). The sensitivity for CV events was 0.691 (0.651–0.730), specificity was 0.531 (0.495–0.566), PPV was 0.505 (0.468–0.541), and NPV was 0.713 (0.675–0.749). The discriminatory power for CV mortality outperformed both the clinical model [AUC 0.707 (0.672–0.741); AUC difference 0.026, *P* = 0.003; NRI 13.4% (0.1–25.8%); IDI 0.019, *P* < 0.001] and the echo model [AUC 0.679 (0.642–0.717); AUC difference 0.054, *P* < 0.001; NRI 15.5% (0.2–27.6%); IDI 0.027, *P* < 0.001]. The discriminatory power for CV events likewise outperformed both the clinical model [AUC 0.625 (0.595–0.656); AUC difference 0.014, *P* = 0.035; NRI 9.7% (0.1–16.6%); IDI 0.021, *P* < 0.001] and the echo model [AUC 0.612 (0.581–0.643); AUC difference 0.028, *P* = 0.019; NRI 10.9% (3.2–18.7%); IDI 0.018, *P* < 0.001].

**Fig. 3:**
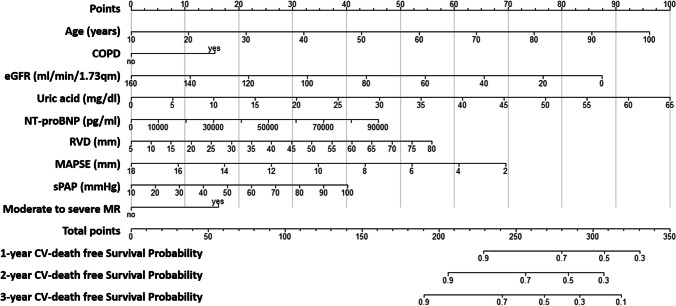
Nomogram based on the C&E risk score designed to predict 1-year, 2-year, and 3-year CV mortality in patients with IHF. This graphical tool offers an intuitive method for estimating the probability of CV mortality by assigning values to each predictor, with each value corresponding to a score on the points scale. The total score is calculated by summing these individual scores. COPD, chronic obstructive pulmonary disease; CV, cardiovascular; eGFR, estimated glomerular filtration rate; IHF, ischemic heart failure; MAPSE, mitral annular plane systolic excursion; MR, mitral regurgitation; NT-proBNP, N-terminal prohormone of brain natriuretic peptide; RVD, end-diastolic mid-right ventricular diameter; sPAP, systolic pulmonary artery pressure

To facilitate the prediction of 1-year, 2-year, and 3-year CV mortality in patients with IHF, we constructed a nomogram based on the C&E risk score. This nomogram offers a straightforward and intuitive means of estimating the probability of CV mortality by assigning values to each predictor, with each value corresponding to a score on the points scale (Fig. 3). The total score of each patient is calculated by summing individual scores of each predictor.

### Risk classification of the IHF patients based on the C&E risk score for CV outcomes prediction

**Fig. 4:**
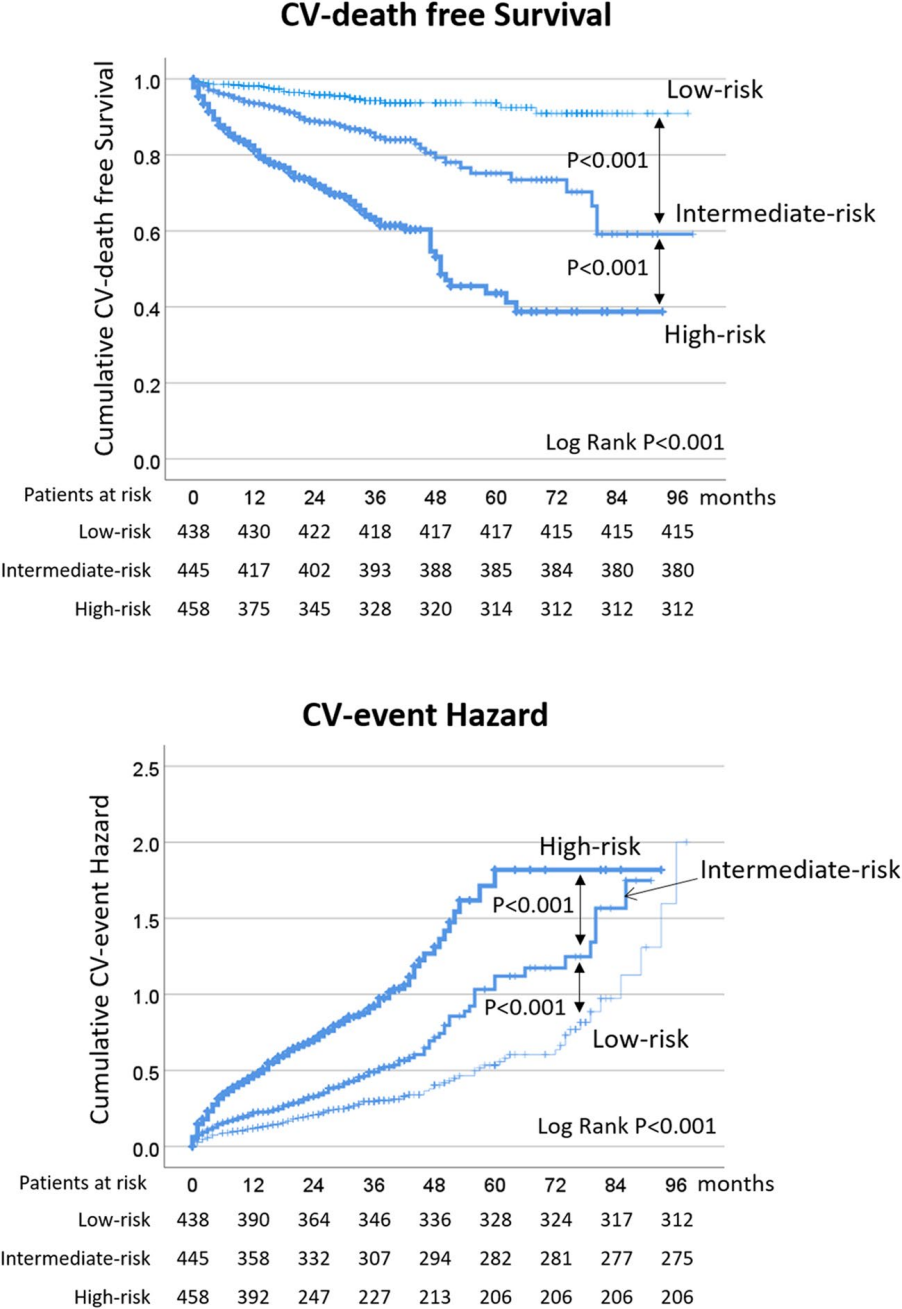
Cumulative CV-death free survival probabilities and CV-event hazard probabilities among low-risk, intermediate-risk, and high-risk patients with IHF through Kaplan–Meier curves. Patients were categorized into low-risk, intermediate-risk, and high-risk groups based on the tertiles of the risk scores within the cohort (low-risk: 0–188, *n* = 438; intermediate-risk: 189–224, *n* = 445; high-risk: 225–350, *n* = 458). The rates of CV mortality (5.3% vs. 14.6% vs. 31.9%) and CV events (28.8% vs. 38.2% vs. 55.0%) exhibit a progressive increase across the low-risk, intermediate-risk, and high-risk groups of patients with IHF and reduced left ventricular ejection fraction (all log rank *P* < 0.001). CV, cardiovascular

The median value of the C&E risk score in this cohort was 208 (180–234) points, and significantly higher in the HFrEF group compared to the HFmrEF group [217 (190–240) vs. 197 (171–223), *P* < 0.001]. Based on tertiles of the risk scores within this cohort, patients were categorized into three risk groups: low-risk (risk score: 0–188, *n* = 438), intermediate-risk (risk score: 189–224, *n* = 445), and high-risk (risk score: 225–350, *n* = 458). The outcomes of interest, including CV mortality (5.3% vs. 14.6% vs. 31.9%, *P* < 0.001), and the rate of CV events (28.8% vs. 38.2% vs. 55.0%, *P* < 0.001), exhibited a progressive increase across low-risk, intermediate-risk, and high-risk patients with IHF. Survival analysis using Kaplan–Meier curves further underscored these distinctions, revealing significant differences in cumulative CV-death free survival probabilities and CV-event hazard probabilities among these risk groups (both log rank *P* < 0.001; Fig. 4).

Comparisons of clinical and echocardiographic characteristics among low-risk, intermediated-risk, and high-risk patients with IHF revealed significant distinctions (Table S1). The high-risk patients were characterized by older age, a higher proportion of women, and an array of clinical factors. Furthermore, they presented with elevated serum levels of creatinine, urea, C-reaction protein, uric acid, and NT-proBNP. High-risk patients also showed a greater proportion of loop diuretics and digoxin use. Conversely, they had lower serum level of eGFR, hemoglobin, and total cholesterol, triglyceride, along with a reduced proportion of angiotensin-converting enzyme inhibitors (ACEIs) or angiotensin II receptor blockers (ARBs) use.

### Adjusted prognostic performance of the C&E risk score for predicting CV outcomes in IHF patients with HFmrEF and HFrEF

The independent prognostic performance of the C&E risk score for predicting CV outcomes was assessed, accounting for potential confounding factors such as age, sex, total cholesterol levels, use of ACEIs or ARBs, loop diuretics, digoxin, E/e´ ratio, and GLS in the analysis (Table 4). After adjusting for these potential confounding factors, the C&E risk score remained as an independent predictor of CV death and CV events in IHF patients. High-risk patients, as determined by the C&E risk score, experienced the most unfavorable CV outcomes. They faced a significantly increased risk of CV mortality (HR 4.567, 95% CI 2.615–7.976, *P* < 0.001), and an increased likelihood of experiencing CV events (HR 2.279, 95% CI 1.698–3.059, *P* < 0.001) compared to the low-risk group, as well as to the intermediate-risk group (CV mortality: HR 2.042, 95% CI 1.479–2.818, *P* < 0.001; CV events: HR 1.694, 95% CI 1.375–2.087, *P* < 0.001) among IHF patients, regardless of whether they fell into the HFmrEF or HFrEF subgroup.

**Table 4:** Adjusted prognostic performance of the C&E risk score for CV outcomes prediction in IHF patients with HFmrEF and HFrEF

	CV mortality			CV events risk		
	Events rate (%)	* Adjusted HR (95% CI)	*P* value	Events rate (%)	* Adjusted HR (95% CI)	*P* value
Total IHF patients (*n* = 1341)						
Intermediate-risk vs. low-risk	14.6 vs. 5.3	2.237 (1.324–3.780)	0.003	38.2 vs. 28.8	1.345 (1.028–3.059)	0.031
High-risk vs. low-risk	31.9 vs. 5.3	4.567 (2.615–7.976)	< 0.001	55.0 vs. 28.8	2.279 (1.698–3.059)	< 0.001
High-risk vs. intermediate-risk	31.9 vs. 14.6	2.042 (1.479–2.818)	< 0.001	55.0 vs. 38.2	1.694 (1.375–2.087)	< 0.001
IHF patients with HFmrEF (*n* = 595)						
Intermediate-risk vs. low-risk	11.7 vs. 5.4	1.557 (0.742–3.267)	0.242	36.5 vs. 25.4	1.263 (0.849–1.878)	0.250
High-risk vs. low-risk	30.4 vs. 5.4	3.722 (1.657–8.358)	0.001	55.8 vs. 25.4	2.090 (1.316–3.318)	0.002
High-risk vs. intermediate-risk	30.4 vs. 11.7	2.391 (1.372–4.166)	0.002	55.8 vs. 36.5	1.655 (1.173–2.335)	0.004
IHF patients with HFrEF (*n* = 746)						
Intermediate-risk vs. low-risk	16.9 vs. 5.1	3.000 (1.392–6.465)	0.005	39.5 vs. 33.7	1.367 (0.953–1.961)	0.090
High-risk vs. low-risk	32.5 vs. 5.1	5.721 (2.589–12.643)	< 0.001	54.7 vs. 33.7	2.436 (1.683–3.525)	< 0.001
High-risk vs. intermediate-risk	32.5 vs. 16.9	1.907 (1.291–2.817)	0.001	54.7 vs. 39.5	1.782 (1.378–2.304)	< 0.001

*Adjusted for age, sex, total cholesterol, use of ACEIs/ARBs, loop diuretics, and digoxin, E/e´ ratio, and GLS

Abbreviations as in the Table 1

### Internal validation of the C&E risk score

Through internal validation employing 1000 bootstrap replicates, the determined median AUC of the C-index based ROC curves was 0.740 (95% CI 0.709 to 0.775, *P* < 0,001) for CV mortality prediction, and 0.678 (95% CI 0.642 to 0.696, *P* < 0.001) for CV events prediction. These results confirms the effective and stable discriminative capability of the C&E risk score in predicting CV mortality and CV events (Figure S1-A). The calibration curve further demonstrated that the nomogram model’s predictions was closely aligned with the actual observations, attesting to the reliability and accuracy of the C&E risk score in predicting CV mortality and CV events (Figure S1-B).

### External validation of the C&E risk score

External validation also confirmed the prognostic efficacy of the established C&E risk score. Consistent predictive performance was observed for 1- and 3-year CV mortality, as well as 3-year CV events risk, not only in the original training dataset but also across diverse validation sets (Figures S2 and S3). This suggests the potential generalizability of the developed risk score for risk stratification among other HF patient populations (Table S2). Additionally, the risk classification of IHF based on the C&E risk score, categorized into low, intermediate, and high-risk groups, demonstrated predictive value for CV mortality and CV events within the validation sets (Figure S4).

### Comparison of the C&E risk score with established Echo Heart Failure Score

We conducted a comparative analysis of the discrimination abilities between the C&E risk score and the published Echo Heart Failure Score (EHFS) [20] for predicting CV mortality in the development cohort (Figure S5). The EHFS incorporates five echocardiographic variables: end-systolic LV volume index ≥ 45 ml/m^2^, LAVi ≥ 84 ml/m^2^, mitral E-wave deceleration time ≤ 140 ms, TAPSE < 16 mm, and sPAP ≥ 45 mmHg, aiming to enhance risk prediction of death in systolic HF patients (LVEF < 45%). In the development cohort, the AUC of the C&E risk score for CV mortality prediction (AUC 0.733) was found to be significantly higher than that for EHFS (AUC 0.667; AUC difference 0.066 (95% CI 0.033–0.100), *P* < 0.001). This comparison underscores the superior discrimination ability of the C&E risk score in predicting CV mortality among IHF patients with LVEF < 50%.

## Discussion

In this study, we established and validated a comprehensive prediction model for CV mortality and CV events risk in IHF patients with LVEF < 50% — C&E risk score. This score incorporates both clinical and echocardiographic predictors, including age, COPD, eGFR, uric acid, NT-proBNP, RVD, MAPSE, sPAP, and moderate to severe MR. The C&E risk score demonstrates significant discriminatory performance in predicting CV outcome in IHF patients, surpassing the predictive capabilities of either clinical or echocardiographic risk score alone. CV outcomes exhibited a proportional increase in C&E risk score-defined low-risk, intermediate-risk, and high-risk IHF patients with reduced LVEF. Internal and external validations further underscore its stability and the potential generalizability of the C&E risk score for risk stratification among other HF patient populations. This innovative C&E risk score might hold the potential to improve the risk assessment of IHF patients with reduced LVEF, help clinical decision-making of individualized monitoring and therapeutic strategies in these patients.

Several echocardiography-based prognostic scores have been developed for HF populations [20, 21]. Huttin et al. introduced the MEDIA echo score, featuring parameters such as sPAP > 40 mmHg, respiratory variation in inferior vena cava diameter > 0.5, E/e´ ratio > 9, and lateral mitral annular s´ < 7 cm/s. This score focuses on predicting all-cause mortality or cardiovascular readmission in HF patients with preserved LVEF (> 50%) [21]. The EHFS introduced by Carluccio et al. incorporating five echocardiographic variables, provides a simple stratification score strongly predictive of the risk of death in patients with chronic systolic HF (LVEF < 45%) [20]. Nevertheless, our findings demonstrate that for IHF patients with reduced systolic function (LVEF < 50%), the developed C&E risk score exhibits better prognostic performance than the EHFS. Stevens et al. reported the value of an echocardiographic score that incorporates five independent echocardiographic predictors, including left ventricular mass index, LAVi, MR, left ventricular outflow tract velocity–time integral, and diastolic dysfunction on predicting the subsequent development of HF in patients with stable coronary artery disease [22]. In general, there is still a knowledge gap in the assessment of CV-related adverse outcomes using an echocardiography-based scoring system in IHF patients with reduced LVEF. This clinical subgroup represents individuals at a notably high risk for HF. Furthermore, the incremental value of adding clinical parameters into the echocardiography-based risk score models for CV outcomes within this specific high-risk cohort of HF patients remains largely unexplored. In present study, we developed a comprehensive C&E risk score focused for IHF patients with reduced LVEF. Besides echocardiographic risk markers, clinical risk indicators, including age, COPD, eGFR, uric acid levels, and NT-proBNP were included in this score. These clinical indicators have previously been established as critical prognostic factors in ischemic heart disease [23, 24]. Our data unequivocally demonstrate that this combined model exhibits significantly enhanced discriminative capability for predicting adverse CV outcomes when compared to either clinical model or echocardiographic model. This novel C&E risk score might hold promise for improving risk assessment and management in these high-risk IHF patients.

The C&E risk score incorporates four key echocardiographic parameters (RVD, sPAP, MAPSE, and moderate to severe MR), each playing a distinct role in assessing cardiac function and providing valuable predictive information. RVD is indicative of RV dysfunction. Our dataset presents compelling evidence suggesting that the mid-cavity diameter of the RV stands out as the most robust predictor of CV mortality. sPAP offers a comprehensive assessment of pulmonary hypertension and cardiac function. Stern et al. reported an association between elevated sPAP (> 50 mmHg) and an increased risk of 1-year HF hospitalizations or all-cause mortality among patients undergoing cardiac resynchronization therapy [25]. The presence of moderate to severe functional MR is associated with significant LV remodeling and dysfunction, offering independent prognostic information in patients with ischemic LV dysfunction [26]. Research conducted by Rossi et al. suggests that severe functional MR can provide clinically relevant information irrespective of left ventricular function, extending its prognostic utility to both ischemic and non-ischemic dilated cardiomyopathy and varying degrees of HF severity [27]. MAPSE is indicative of global longitudinal function of the left ventricle [28, 29] and can be utilized to evaluate contractile reserve in patients with ischemic cardiomyopathy [30]. MAPSE has demonstrated its independence as a predictor of adverse outcomes across diverse patient populations [31–33]. It is noteworthy that GLS is a newer and more refined echocardiographic measure for assessing LV longitudinal function [34]. Clinical investigations have underscored the prognostic significance of GLS in a range of cardiovascular conditions [35], including chronic ischemic cardiomyopathy [36] and ST-segment elevation myocardial infarction [37]. Nonetheless, our analysis yielded an intriguing finding. Through the variable selection procedure, GLS did not emerge as a retained feature in our model. This may be attributed to GLS serving as an integrative parameter that potentially overlaps with other systolic function variables; further investigation is needed to clarify this issue.

### Clinical implications

Our study holds key clinical implications. The developed C&E risk score, with its analytical edge, integrates diverse risk factors into a comprehensive model, surpassing the predictive capability of individual indicators. Although the AUC values fall below the threshold of 0.8, indicative of moderate predictive performance, the C&E risk score surpasses both clinical and echocardiographic models in predicting CV outcomes for this patient population. Internal and external validations have consistently demonstrated the stability and good generalizability of the risk stratification offered by the C&E score. This means that the score effectively identifies high-risk IHF patients, enabling proactive measures such as intensified monitoring and aggressive therapeutic interventions. Patients were categorized into low-, intermediate-, and high-risk groups based on tertiles of their risk scores. Our results confirm the utility of this grouping for risk stratification. Present findings demonstrate that this risk classification strategy aligns with clinical outcomes, effectively distinguishing between patients at varying risk levels. This approach might simplify communication for healthcare professionals and facilitate practical implications for tailoring interventions based on identified risk groups. Overall, our study supports the validity and utility of tertile-based risk classification as a valuable tool for personalized patient management.

### Study limitations

Our study primarily focused on IHF patients with LVEF < 50%, limiting the generalizability of our findings to IHF patients with HFpEF. The data used in our retrospective analysis were derived from a single-center study, which may be related to selection bias and future external validity study is needed to verify our findings. Prospective studies are warranted to see if individualized therapy option could improve the CV outcome of high-risk IHF patients as defined by this score. Echocardiographic measurements vary among different operators and institutions, potentially affecting the accuracy and consistency of the C&E risk score. Standardized imaging protocols and quality control measures are thus essential to minimize the influence from these factors. Additionally, initial data collection did not include relevant information about coronary heart disease, such as angiography data and cardiac enzymology. Furthermore, we failed to obtain an independent cohort from another hospital or published studies with consistent MASPE measurements for validation. Future studies are warranted to further explore and clarify this issue. In light of this limitation, the validation was conducted in newly hospitalized IHF patients within our hospital in 2018.

It is crucial to acknowledge that the enrolled patients in our study experienced relatively high mortality rates and did not receive contemporary HF therapies in accordance with current guidelines. Approximately 8% of IHF patients in the development cohort received ARNIs, and none was prescribed sodium-glucose co-transporter 2 (SGLT2) inhibitors at baseline. The limited adoption of modern HF therapies raises valid concerns regarding the generalizability of our findings. Caution is warranted when interpreting mortality and associated outcomes due to the restricted use of advanced HF medications within our cohort. The retrospective nature of the data restricts our ability to assess the impact of initiating or switching to contemporary HF treatments on the C&E risk score. Notably, when compared to the development cohort (enrolled before 2018), CV mortality in IHF patients treated with ARNIs (validation set 2) was significantly lower (10.2% vs. 17.4%, *P* = 0.016), suggesting improved outcomes with the application of new HF medication. Nevertheless, the C&E risk score consistently demonstrated prognostic ability for CV mortality in this validation set, indicating potential applicability in the modern era of HF management. Prospective studies, including patients receiving modern HF therapies, are warranted to provide nuanced insights into the predictive performance of the C&E risk score in the evolving landscape of HF treatment strategies.

## Conclusions

This study establishes and validates the novel C&E risk score as a reliable tool for predicting CV outcomes in IHF patients with reduced LVEF. The risk score holds potential for enhancing risk stratification and guiding clinical decision-making for high-risk patients.

## Supplementary information

Below is the link to the electronic supplementary material.

Supplementary file1 (TIF 4633 KB)

Supplementary file2 (TIF 5333 KB)

Supplementary file3 (TIF 5391 KB)

Supplementary file4 (TIF 5253 KB)

Supplementary file5 (TIF 3998 KB)

Supplementary file6 (DOCX 44 KB)

Supplementary file7 (XLSX 814 KB)

Supplementary file8 (XLSX 101 KB)

Supplementary file9 (XLSX 169 KB)

Supplementary file10 (XLSX 102 KB)

## Data Availability

The data that support the findings of this study are available on request from the corresponding author (PN). The data are not publicly available due to their containing information that could compromise the privacy of research participants.
