# Supplementary figures and images for: Clinical value of a comprehensive clinical- and echocardiography-based risk score on predicting cardiovascular outcomes in ischemic heart failure patients with reduced ejection fraction
Source: Clin Res Cardiol. 2024 Mar 6;114(5):541–56. doi: 10.1007/s00392-024-02399-1 (PMC12058811; doi:10.1007/s00392-024-02399-1)

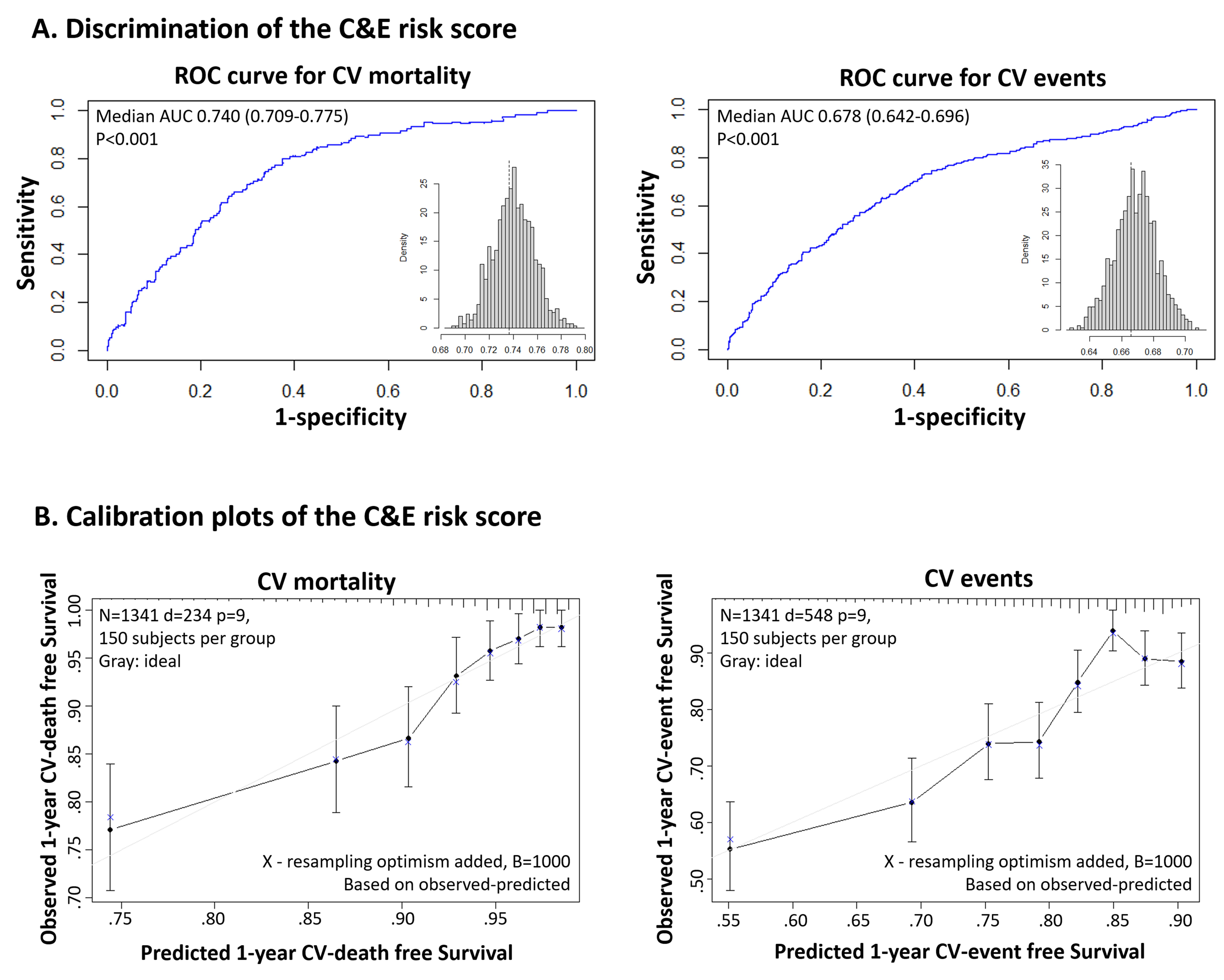

Supplement: Supplementary file 1 — Supplementary file1 (TIF 4633 KB) [file 392_2024_2399_MOESM1_ESM.tif]

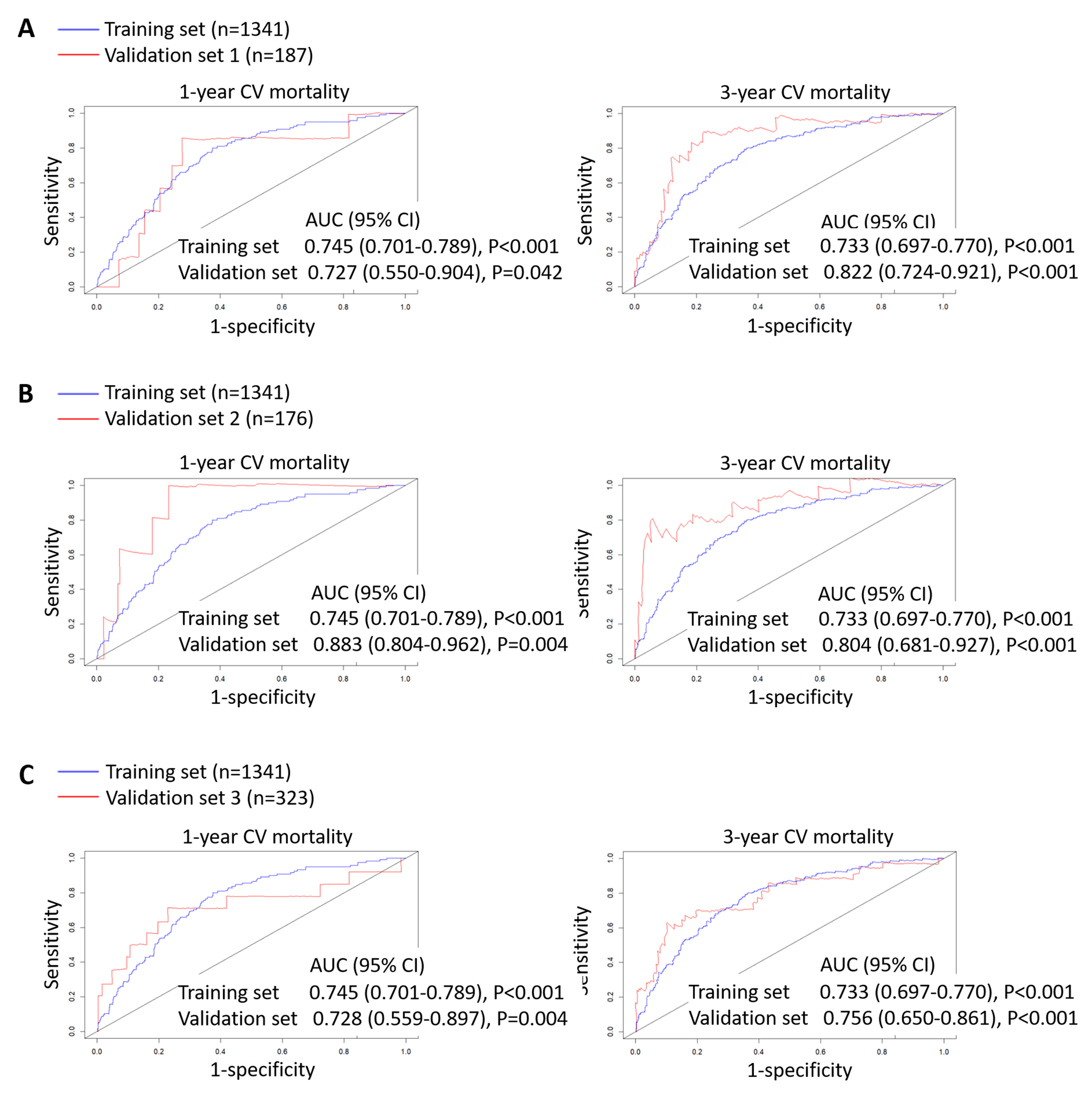

Supplement: Supplementary file 2 — Supplementary file2 (TIF 5333 KB) [file 392_2024_2399_MOESM2_ESM.tif]

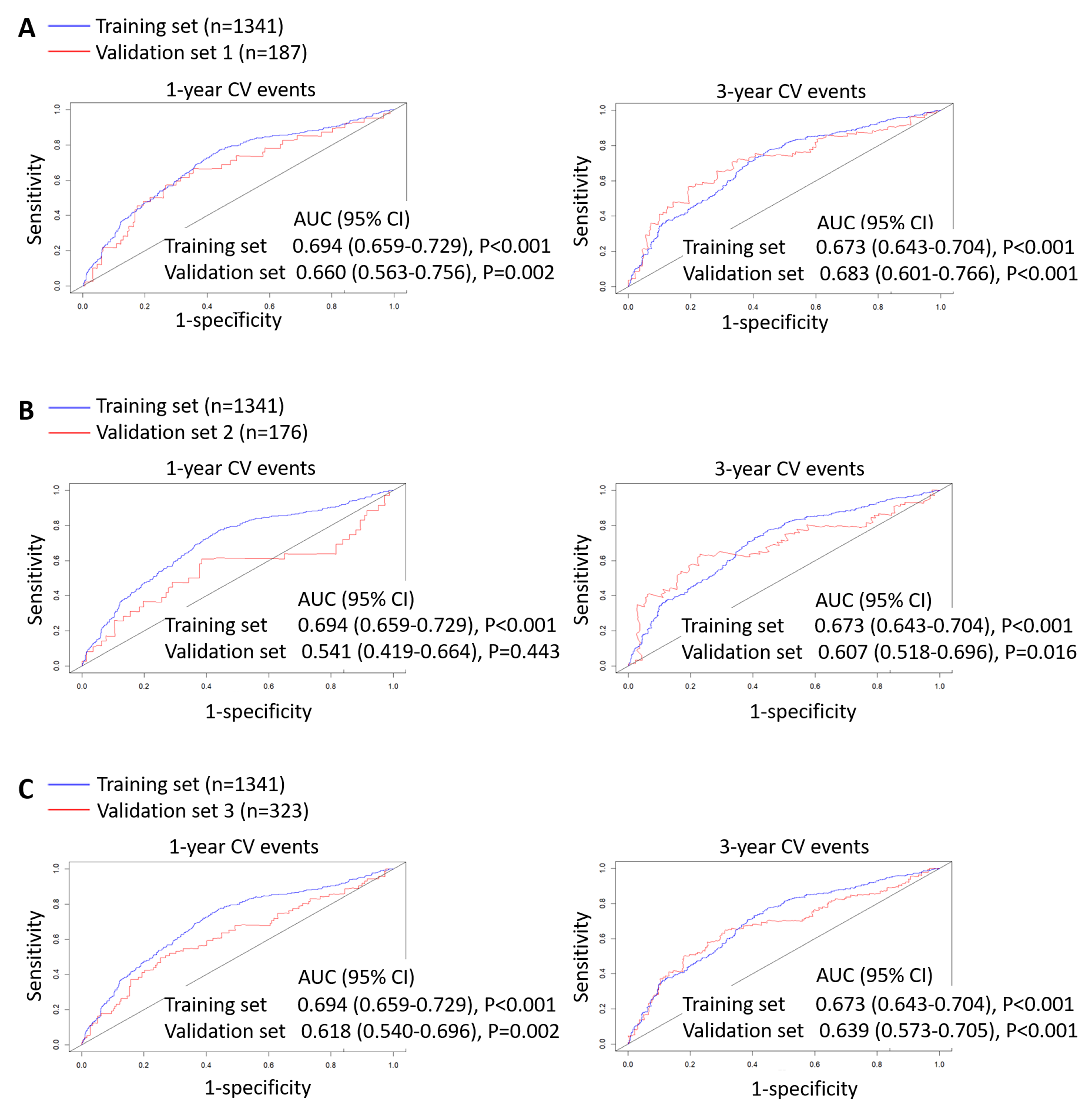

Supplement: Supplementary file 3 — Supplementary file3 (TIF 5391 KB) [file 392_2024_2399_MOESM3_ESM.tif]

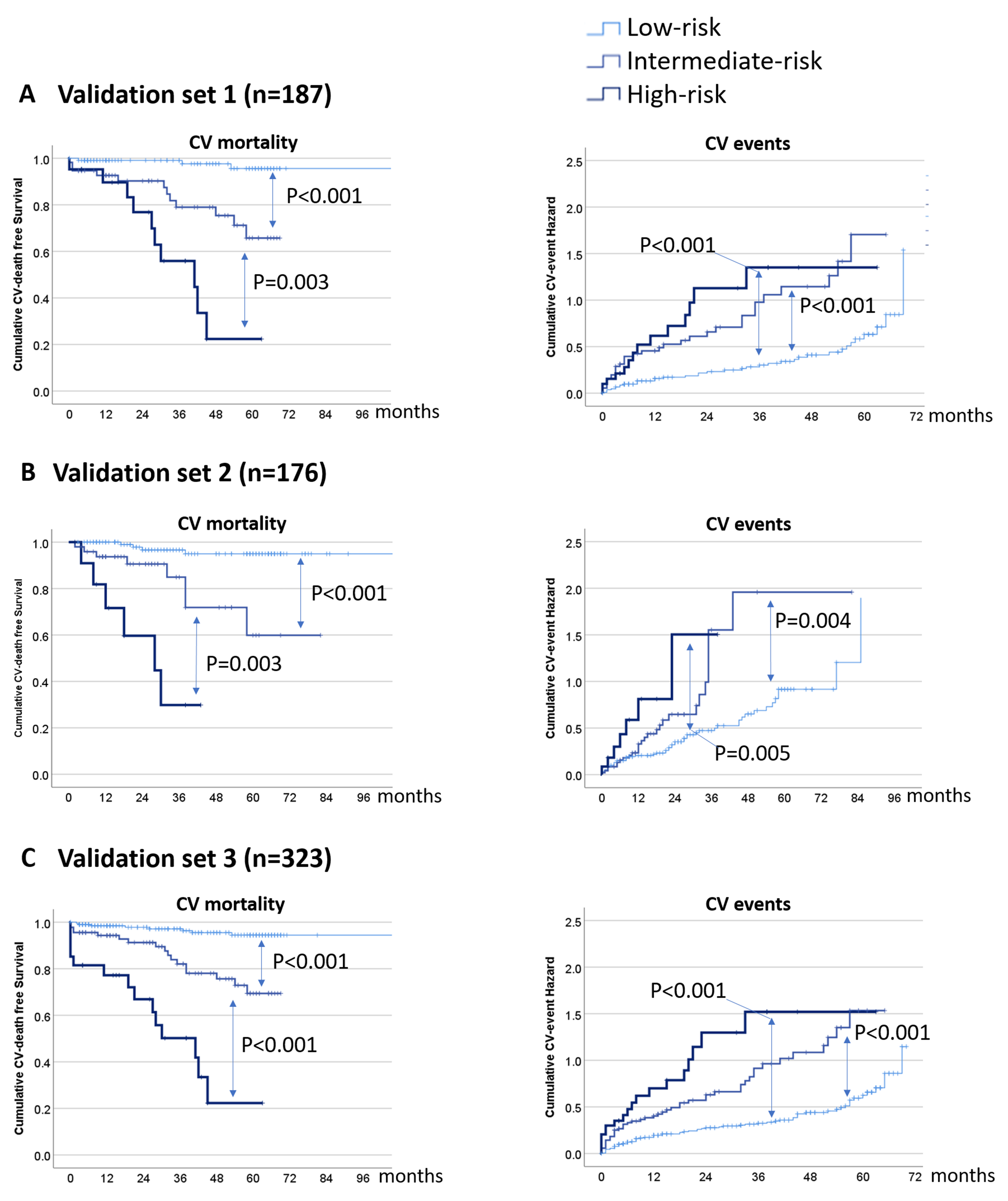

Supplement: Supplementary file 4 — Supplementary file4 (TIF 5253 KB) [file 392_2024_2399_MOESM4_ESM.tif]

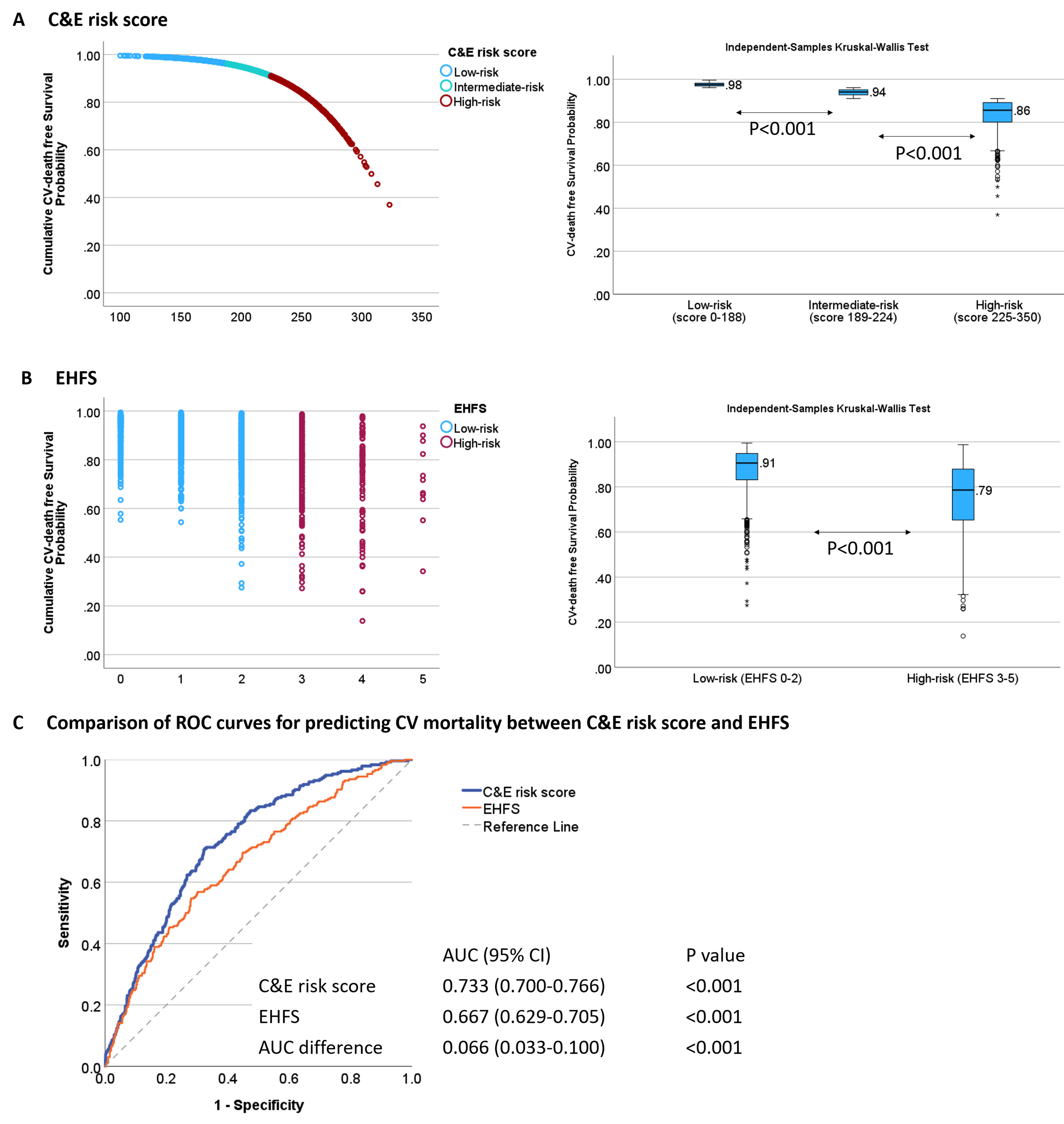

Supplement: Supplementary file 5 — Supplementary file5 (TIF 3998 KB) [file 392_2024_2399_MOESM5_ESM.tif]
