# Supplementary material for: Clinical value of a comprehensive clinical- and echocardiography-based risk score on predicting cardiovascular outcomes in ischemic heart failure patients with reduced ejection fraction
Source: Clin Res Cardiol. 2024 Mar 6;114(5):541–56. doi: 10.1007/s00392-024-02399-1 (PMC12058811; doi:10.1007/s00392-024-02399-1)
Supplement: Supplementary file 6 — Supplementary file6 (DOCX 44 KB) [file 392_2024_2399_MOESM6_ESM.docx]

**Supplementary Tables**

**Table S1 Clinical characteristics and outcomes stratified by the C&E risk score classification**

|  | Low-risk | Intermediate-risk | High-risk | P value |
| --- | --- | --- | --- | --- |
| No. | 438 (32.7) | 445 (33.2) | 458 (34.2) |  |
| Clinical outcomes |  |  |  |  |
| Follow-up duration (months) | 33 (19-45) | 27 (15-39)* | 20 (10-32)*† | <0.001 |
| CV mortality [n (%)] | 23 (5.3) | 65 (14.6)* | 146 (31.9)*† | <0.001 |
| CV hospitalization [n (%)] | 110 (25.1) | 135 (30.3) | 179 (39.1)*† | <0.001 |
| Combined CV events [n (%)] | 126 (28.8) | 170 (38.2)* | 252 (55.0)*† | <0.001 |
| Age (years) | 60.6±10.1 | 72.8±8.8* | 77.1±8.6*† | <0.001 |
| Male [n (%)] | 366(83.6) | 353 (79.3) | 348 (76.0)* | 0.019 |
| Body mass index [kg/m²] | 28.1±4.8 | 28.0±4.6 | 26.7±4.4*† | <0.001 |
| NYHA class III-IV [n (%)] | 81 (18.5) | 135 (30.3)* | 237 (51.7)*† | <0.001 |
| Systolic blood pressure (mmHg) | 129±23 | 132±24 | 128±25† | 0.024 |
| Diastolic blood pressure (mmHg) | 74±14 | 74±15 | 73±14* | 0.018 |
| Angina pectoris [n (%)] | 135 (30.8) | 117 (26.3) | 155 (33.8)† | 0.046 |
| Prior myocardial infarction [n (%)] | 311 (71.0) | 250 (56.2)* | 255 (55.7)* | <0.001 |
| PCI [n (%)] | 277 (63.2) | 239 (53.7)* | 220 (480)* | <0.001 |
| CABG [n (%)] | 84 (19.2) | 146 (32.8)* | 151 (33.0)* | <0.001 |
| Comorbidities [n (%)] |  |  |  |  |
| Atrial fibrillation | 64 (14.6) | 119 (26.7)* | 203 (44.3)*† | <0.001 |
| Obesity | 173 (39.5) | 184 (41.3) | 159 (34.7) | 0.107 |
| Hypertension | 290 (66.2) | 347 (78.0)* | 357 (77.9)* | <0.001 |
| Diabetes | 113 (25.8) | 182 (40.9)* | 183 (40.0)* | <0.001 |
| Hyperlipidemia | 207 (47.3) | 181 (40.7) | 162 (35.4)* | 0.001 |
| Smoking status |  |  |  | <0.001 |
| Never smoked | 208 (47.5) | 312 (70.1)* | 330 (72.1)* |  |
| Ex-smoking | 92 (21.0) | 78 (17.5) | 82 (17.9) |  |
| Currently smoking | 138 (31.5) | 55 (12.4)* | 46 (10.0)* |  |
| Hyperuricemia | 79 (18.0) | 188 (42.2)* | 288 (62.9)*† | <0.001 |
| Anemia | 205 (46.8) | 278 (62.5)* | 322 (70.3)*† | <0.001 |
| Renal dysfunction | 26 (5.9) | 194 (43.6)* | 370 (80.8)*† | <0.001 |
| Stroke / TIA | 27 (6.2) | 46 (10.3) | 60 (13.1)* | 0.002 |
| Peripheral vascular disease | 28 (6.4) | 51 (11.5)* | 65 (14.2)* | <0.001 |
| COPD | 29 (6.6) | 50 (11.2)* | 100 (21.8)*† | <0.001 |
| Sleep disorders | 21 (4.8) | 23 (5.2) | 39 (8.5) | 0.038 |
| ICD / CRT-D implantation [n (%)] | 63 (14.4) | 87 (19.6) | 95 (20.7)* | 0.033 |
| Laboratory data (median, IQR) |  |  |  |  |
| eGFR (ml/min/1.73qm) | 85 (74-97) | 63 (51-76)* | 44 (29-56)*† | <0.001 |
| Creatinine (mg/dl) | 0.92 (0.80-1.05) | 1.14 (0.99-1.37)* | 1.53 (1.22-2.15)*† | <0.001 |
| Urea (mg/dl) | 32.2 (26.6-39.1) | 42.7 (33.6-57.0)* | 63.7 (46.8-92.5)*† | <0.001 |
| C-reaction protein (mg/dl) | 0.83 (0.25-2.75) | 0.78 (0.22-2.52)* | 1.51 (0.52-3.66)*† | <0.001 |
| Uric acid (mg/dl) | 5.7 (4.8-6.9) | 6.7 (5.6-8.2)* | 8.0 (6.3-10.0)*† | <0.001 |
| Hemoglobin (g/dl) | 13.8 (12.4-14.8) | 13.0 (11.8-14.1)* | 12.0 (10.6-13.7)*† | <0.001 |
| Cholesterol (mg/dl) | 169 (144-199) | 162 (137-189)* | 151 (127-188)*† | <0.001 |
| Triglyceride (mg/dl) | 129 (98-179) | 124 (98-172) | 113 (85-156)*† | <0.001 |
| HDLC (mg/dl) | 40 (32-51) | 45 (35-54)* | 41 (32-52)† | <0.001 |
| LDLC (mg/dl) | 98 (73-121) | 88 (69-110)* | 84 (62-111)* | <0.001 |
| NT-proBNP (pg/ml) | 963 (378-2399) | 2357 (1075-5257)* | 6814 (2444-15740) *† | <0.001 |
| hsTnT (pg/ml) | 125.4 (21.2-1517.0)  n=195 | 60.4 (23.7-357.5)  n=191 | 61.9 (37.4-188.4)  n=169 | 0.231 |
| Medications [n (%)] |  |  |  |  |
| Beta-blockers | 376 (85.8) | 375 (84.3) | 391 (85.4) | 0.795 |
| ACEIs / ARBs | 382 (87.2) | 381 (85.6) | 336 (73.4)*† | <0.001 |
| MRAs | 150 (34.2) | 145 (32.6) | 127 (27.7) | 0.091 |
| Loop diuretics | 140 (32.0) | 270 (60.7)* | 369 (80.6)*† | <0.001 |
| Digoxin | 25 (5.7) | 39 (8.8) | 89 (19.4)† | <0.001 |

Data are expressed as mean ± standard deviation, median with interquartile range (Q1-Q3), or as number (%).

* P<0.05 vs. low-risk group, † P<0.05 vs. intermediate-risk group.

ACEIs, angiotensin-converting enzyme inhibitors; ARBs, angiotensin II receptor antagonists; CABG, coronary artery bypass grafting; COPD, chronic obstructive pulmonary disease; CRT-D, cardiac resynchronization therapy with defibrillator; CV, cardiovascular; E/e´ ratio, the ratio of early diastolic mitral inflow velocity to mitral annular tissue velocity; eGFR, estimated glomerular filtration rate; GLS, global longitudinal strain; HDLC, high-density lipoprotein cholesterol; hsTnT, high-sensitive troponin T; ICD, implantable cardioverter defibrillator; IVSd, end‐diastolic interventricular septal thickness; LAVi, left atrial volume indexed to body surface area; LDLC, low-density lipoprotein cholesterol; LVEDD, left ventricular end‐diastolic dimension; LVEF, left ventricular ejection fraction; LVPWd, end‐diastolic posterior wall thickness; MAPSE, mitral annular plane systolic excursion; MR, mitral regurgitation; MRAs, mineralocorticoid receptor antagonists; NT-proBNP, N-terminal prohormone of brain natriuretic peptide; NYHA, New York Heart Association; PCI, percutaneous coronary intervention; RAA, end‐systolic right atrial area; RVD, end‐diastolic mid‐right ventricular diameter; sPAP, systolic pulmonary artery pressure; TAPSE, tricuspid annular plane systolic excursion; TIA, transient ischemic attack; TR, tricuspid regurgitation.

**Table S2 Clinical and echocardiographic characteristics among the training set and three different validation sets**

|  | Training set | Validation set 1 | Validation set 2 | Validation set 3 | P value |
| --- | --- | --- | --- | --- | --- |
| Source | IHF hospitalized from 2009 to 2017 | IHF hospitalized in 2018 | IHF treated with ARNI therapy hospitalized between 2016 and 2018 | A combined cohort of IHF and non-IHF hospitalized in 2018 |  |
| No. | 1341 | 187 | 176 | 323 |  |
| Age (years) | 70±11 | 73±10* | 65±12*† | 70±12†‡ | <0.001 |
| Male [n (%)] | 1067 (79.6) | 138 (73.8) | 146 (83.0) | 221 (68.4)*‡ | <0.001 |
| NYHA class III-IV [n (%)] | 453 (33.8) | 98 (52.4) | 79 (44.9) | 168 (52.0) | <0.001 |
| COPD [n (%)] | 179 (13.3) | 24 (12.8) | 23 (13.1) | 32 (9.9) | 0.424 |
| eGFR (ml/min/1.73qm) | 64 (47-81) | 60 (44-80) | 65 (45-83) | 60 (42-79) | 0.068 |
| Uric acid (mg/dl) | 6.7 (5.3-8.4) | 6.8 (5.1-8.7) | 7.5 (5.8-9.0)* | 6.8 (5.5-8.6) | 0.012 |
| NT-proBNP (pg/ml) | 2412 (904-7085) | 3121 (1226-8604) | 2546 (875-6086) | 3072 (1179-8180) | 0.012 |
| RVD (mm) | 28.0 (22.0-33.0) | 28.0 (24.0-33.0) | 30.0 (24.0-35.0) | 29.0 (25.0-34.0) | 0.008 |
| MAPSE(mm) | 8.0 (6.5-10.0) | 8.0 (7.0-9.5) | 7.5 (6.0-9.0)* | 8.0 (6.5-9.5) | 0.059 |
| sPAP (mmHg) | 34.0 (26.0-46.0) | 36.0 (28.0-46.9) | 32.4 (26.0-45.0) | 36.0 (28.0-47.0) | 0.039 |
| Moderate to severe MR [n (%)] | 214 (16.0) | 37 (19.8) | 35 (19.9) | 261 (19.2) | 0.248 |
| Clinical outcomes [n (%)] |  |  |  |  |  |
| Death | 376 (28.0) | 33 (17.6)* | 22 (12.5)* | 49 (15.2)* | <0.001 |
| Heart transplantation | 5 (0.4) | 0 (0.0) | 0 (0.0) | 2 (0.6) |  |
| CV mortality | 234 (17.4) | 25 (13.4) | 18 (10.2) | 39 (12.1) | 0.011 |
| Combined CV events | 548 (40.9) | 88 (47.1) | 86 (48.9) | 144 (44.6) | 0.090 |

Data are expressed as mean ± standard deviation, median with interquartile range (Q1-Q3), or as number (%).

Significance values were adjusted by the Bonferroni correction for multiple tests.

* P<0.05 vs. training set, † P<0.05 vs. validation set 1, ‡ P<0.05 vs. validation set 2

Abbreviations as in the Table S1.
